# Feasibility Theory Reconciles and Informs Alternative Approaches to Neuromuscular Control

**DOI:** 10.3389/fncom.2018.00062

**Published:** 2018-09-11

**Authors:** Brian A. Cohn, May Szedlák, Bernd Gärtner, Francisco J. Valero-Cuevas

**Affiliations:** ^1^Department of Computer Science, University of Southern California, Los Angeles, CA, United States; ^2^Department of Theoretical Computer Science, ETH Zurich, Zurich, Switzerland; ^3^Department of Biomedical Engineering, University of Southern California, Los Angeles, CA, United States; ^4^Division of Biokinesiology and Physical Therapy, University of Southern California, Los Angeles, CA, United States

**Keywords:** feasibility, neuromechanics, motor control, tendon-driven, dimensionality, synergies, optimization, forces

## Abstract

We present Feasibility Theory, a conceptual and computational framework to unify today's theories of neuromuscular control. We begin by describing how the musculoskeletal anatomy of the limb, the need to control individual tendons, and the physics of a motor task uniquely specify the family of all valid muscle activations that accomplish it (its ‘feasible activation space’). For our example of producing static force with a finger driven by seven muscles, computational geometry characterizes—in a complete way—the structure of feasible activation spaces as 3-dimensional polytopes embedded in 7-D. The feasible activation space for a given task is *the* landscape where all neuromuscular learning, control, and performance must occur. This approach unifies current theories of neuromuscular control because the structure of feasible activation spaces can be separately approximated as either low-dimensional basis functions (synergies), high-dimensional joint probability distributions (Bayesian priors), or fitness landscapes (to optimize cost functions).

## 1. Introduction

**Figure 1:**
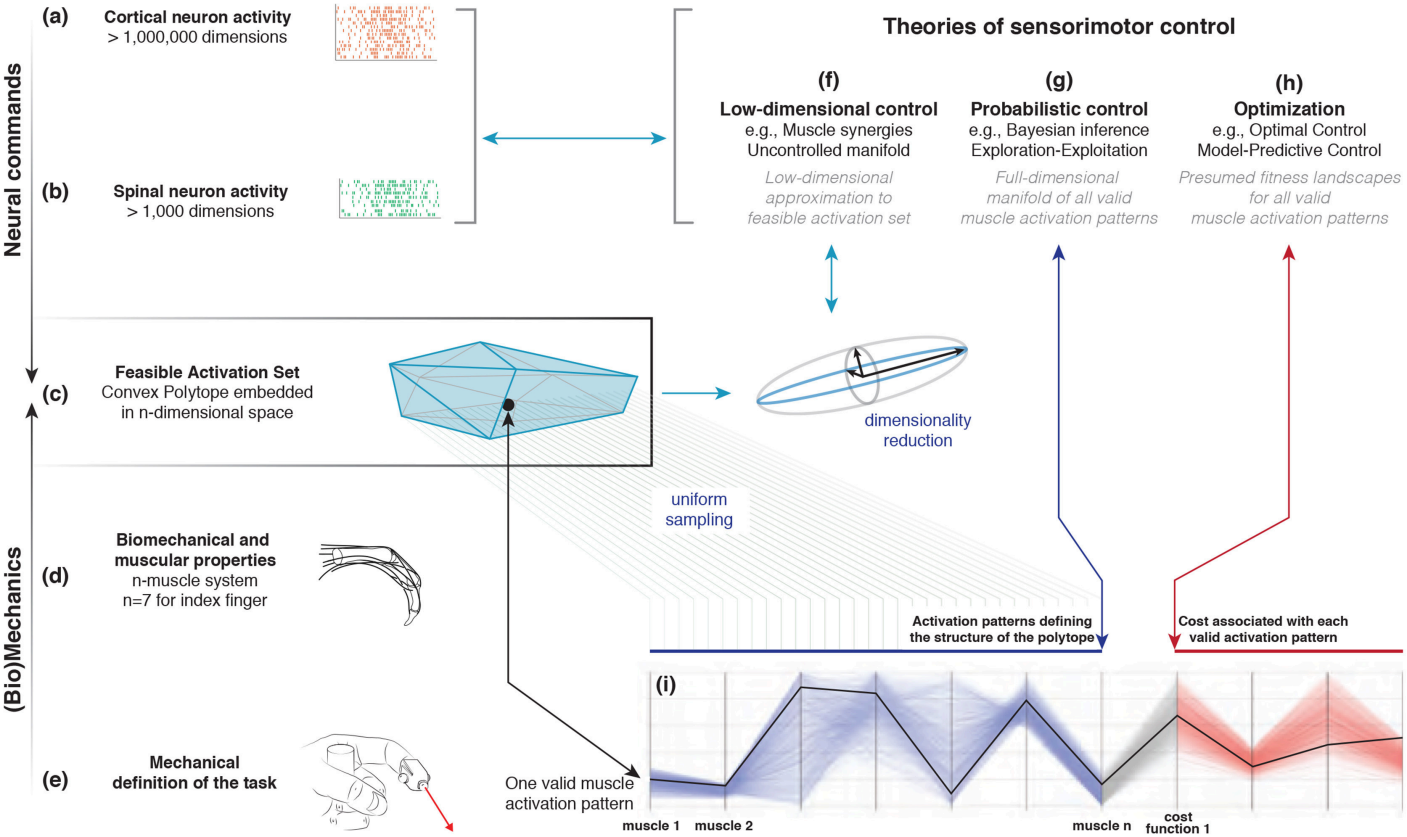
Emergence and interpretation of feasible activation spaces for a particular motor task. The descending motor command for a given task is issued by the motor cortex **(a)**, which projects onto inter-neurons and *alpha*-motor neuron pools in the spinal cord **(b)**. The combined drive to all *alpha*-motor neurons of a muscle can be considered its total muscle activation level (a value between 0 and 1). If we consider that muscles can, to a large extent, be controlled independently and in different ways, then the overall motor command can be conceptualized as a multi-dimensional *muscle activation pattern* (i.e., a point) in a high-dimensional *muscle activation space* (Chao and An, 1978; Spoor, 1983; Kuo and Zajac, 1993; Valero-Cuevas et al., 1998; Todorov and Jordan, 2002) **(c)**. For that muscle activation pattern to be valid, it has to elicit muscle forces **(d)** capable of satisfying the mechanical constraints of the task—in this case defining a well-directed sub-maximal fingertip force **(e)**. Given the large number of muscles in vertebrates, there can be muscle redundancy: where a given task can be accomplished with a large number of valid muscle activation patterns. We propose that our novel ability to characterize the high-dimensional structure of feasible activation spaces **(i)** allows to us to compare, contrast, and reconcile today's three dominant approaches to muscle redundancy in sensorimotor control **(f–h)**.

How the nervous system selects specific levels of muscle activations (i.e., a muscle activation pattern) for a given motor task continues to be hotly debated. Some suggest the nervous system either combines low-dimensional synergies (Dingwell et al., 2010; Kutch and Valero-Cuevas, 2012; Alessandro et al., 2013; Bizzi and Cheung, 2013; Rácz and Valero-Cuevas, 2013; Steele et al., 2013, 2015), learns probabilistic representations of valid muscle activation patterns (Körding and Wolpert, 2004; Sanger, 2011; Berniker et al., 2013; Kording, 2014), or optimizes physiologically-tenable cost functions (Chao and An, 1978; Crowninshield and Brand, 1981; Prilutsky, 2000; Todorov and Jordan, 2002; Scott, 2004; Higginson et al., 2005). At the core of this problem lies the nature of “feasible activation spaces,” and the computational challenge of describing and understanding their high-dimensional structure (for an overview, see Valero-Cuevas, 2015). A feasible activation space is the family of valid solutions (i.e., muscle activation patterns) that meet the mechanical constraints^1^ of a given motor task. Figure 1 illustrates these neuromechanical interactions that define the feasible activation space for a particular task.

The most the nervous system can do, therefore, is select and apply a specific muscle activation pattern from within the feasible activation space. This is because muscle activation patterns outside of this space are, by definition, inappropriate for the task. In fact, the feasible activation space defines the landscape upon which all neuromuscular learning and performance must occur for that task. Studying neuromuscular control is, therefore, equivalent to studying how the nervous system finds, explores, inhabits, and exploits the contents and structure of feasible activation spaces (Dingwell et al., 2010; Kutch and Valero-Cuevas, 2012; Bizzi and Cheung, 2013; Rácz and Valero-Cuevas, 2013; Steele et al., 2013, 2015; Gallego et al., 2017).

But the “curse of dimensionality” (Bellman and Osborn, 1958; Avis and Fukuda, 1992; Bellman, 2015) makes it computationally challenging to calculate, describe, and understand the nature and structure of high-dimensional feasible activation spaces (Chao and An, 1978; Spoor, 1983; Kuo and Zajac, 1993; Scholz and Schöner, 1999; Valero-Cuevas et al., 2009a; Dingwell et al., 2010; Theodorou and Valero-Cuevas, 2010)—even for an isolated human finger or cat leg generating everyday static forces (Kutch and Valero-Cuevas, 2012; Sohn et al., 2013; Valero-Cuevas, 2015; Valero-Cuevas et al., 2015b). This is due to the computational complexity of algorithms to map the geometric details of objects embedded in high dimensions (Smith, 1984; Lovász, 1999; Fukuda, 2014).

Current theories of neuromuscular control^2^ are alternative responses to overcome the curse of dimensionality in this context. These alternative approaches, however, are seldom combined and often the insights from one realm are not readily applicable to the others. Here we emphasize how the mechanics of the body and the physics of the task constitute the common ground for all theories.

We now propose “Feasibility Theory,” which is a conceptual framework to characterize feasible activation spaces in detail. While prior work has described *how* to find such feasible activation spaces for static force production (Valero-Cuevas et al., 1998, 2015a; Venkadesan and Valero-Cuevas, 2008; Kutch and Valero-Cuevas, 2012; Marjaninejad and Valero-Cuevas, 2019), we now explain *why* the structure of a feasible activation space can be approximated with low-dimensional synergies and probability distribution functions, and can be associated with multiple fitness landscapes over which to optimize (Table 1).

**Table 1:** Applicability and compatibility of Feasibility Theory with dominant theories of neuromuscular control.

**Dimensionality Reduction**	**PCA, NMF, etc. describe the general shape and structure of the feasible activation space. The resulting basis functions serve as an approximation of the input-output relationship of the system (i.e., descriptive synergies)**.
Motor Primitives / Synergies / Modular Organization	If the basis functions mentioned above are of neural origin, they would be the means by which the nervous system inhabits the feasible activation space and executes valid solutions (i.e., prescriptive synergies).
Uncontrolled Manifold (UCM) Theory	The UCM Theory emphasizes that the temporal evolution of muscle activation patterns in the interior of the feasible activation space need not be as tightly controlled as those at its boundaries. This is because moving between interior points has no impact on the output as they constitute the null-space of the task (i.e., they are “goal-equivalent” as in Scholz and Schöner, 1999). In contrast, Feasibility Theory describes details of the *structure* of the feasible activation space.
Exploration-Exploitation	Heuristic and trial-and-error approaches can be used to find points within the Feasible Activation Space because it is a needle-in-a-haystack problem. By definition, there is a small likelihood of finding a point on a low-dimensional manifold embedded in a high-dimensional space (e.g., the volume of a line is zero). Thus, the families of valid solutions found are preferentially adopted (e.g., as motor habits De Rugy et al., 2012). Such a heavily iterative approach is compatible with reinforcement learning (Valero-Cuevas et al., 2009a), motor babbling (Touwen, 1976), the hundreds of thousands of steps children take when learning to walk (Adolph et al., 2012), or the mass practice a patient needs for effective rehabilitation (Lang et al., 2009).
Probabilistic Neuromuscular Control	If muscle activation patterns within the feasible activation space can be found (by any means), they can be combined to build probability density functions (i.e., Bayesian priors). A likely valid action for a particular situation can then be selected via Bayes' Theorem (e.g., Körding and Wolpert, 2004).
Optimization / Minimal Intervention Principle/ Optimal Control	Every point in the feasible activation space is, by definition, valid. However, if a cost function is used to evaluate each point in it, the feasible activation space is transformed into a fitness landscape. Optimization methods can then navigate this fitness landscape to find local and global minima (e.g., Crowninshield and Brand, 1981; Anderson and Pandy, 2001; Todorov and Jordan, 2002).

## 2. Methods

In the case of the seven muscles of the human index finger producing static fingertip force, we show that the family of feasible commands, the feasible activation space, is a 3-dimensional polytope embedded in 7-dimensional muscle activation space (Valero-Cuevas et al., 1998). A “polytope” is the formal name for bounded polyhedra in dimensions higher than three. With 4 task constraints applied to 7 muscles, the result is a 3-dimensional polytope embedded in the 7-dimensional muscle activation space. By construction of anatomy, producing static force with a fixed posture naturally leads to a relationship between muscle forces and endpoint torques. The linear constraint equations that define this relationship (and in parallel the polytope that arises from the constraints) accurately represent the set of feasible motor commands (Valero-Cuevas et al., 1998; Sohn et al., 2013; Valero-Cuevas, 2015). Our computational approach hinges on the efficient sampling and complete representation of the geometric structure of high-dimensional polytopes which fully characterizes the family of all valid muscle activation patterns–each of which solves the same task. By definition, this polytope is the null space of the task.

The methods to obtain feasible activation spaces for “tendon-driven” limbs are described in detail in the textbook *Fundamentals of Neuromechanics* and references therein (Valero-Cuevas, 2015). This tendon-driven approach explicitly and distinctly avoids the conceptual approach to calculate net torques at each joint. Rather, it emphasizes studying the individual actions of all muscles at all levels of analysis, from their neural activation to their contributions to fingertip force. We describe them briefly here.

Consider a tendon-driven limb, such as a finger, with *n* independently controllable muscles, where we define the neural command to each muscle as a positive value of activation between 0 (no activation) and 1 (maximal activation), where a value of 1 would produce the maximum possible tendon force for that muscle. We do not differentiate between concentric or eccentric contraction—we define muscle activation as the net static tendon tension, normalized by the maximum tendon tension possible by that muscle. We can then visualize the set of all feasible neural commands (i.e., all possible muscle activation patterns) as the points contained in a positive n-dimensional cube with sides of length equal to 1. A specific muscle activation pattern is a *point* (i.e., an n-dimensional vector **a**) in this n-dimensional cube (Chao and An, 1978; Spoor, 1983; Kuo and Zajac, 1993; Valero-Cuevas et al., 1998). Now consider a specific task, such as producing a vector of static force with the fingertip, as when holding an object. Clearly, not all muscle activation patterns inside the n-dimensional cube can produce that desired static fingertip force vector: bone lengths, kinematic degrees of freedon, anatomical routing, posture, and muscle strength inequities define the subset of points in the *n*-cube which produce a fingertip force vector of a specific magnitude and direction. As described in Chao and An (1978), Spoor (1983), Kuo and Zajac (1993), Valero-Cuevas (2015) the musculoskeletal anatomy of the limb, the need to control individual tendons, and the physics of a motor task uniquely specify a polytope embedded in ℝ^*n*^ (i.e., the feasible activation space). This polytope contains the family of (potentially infinite) valid muscle activation patterns that can produce this static force production task. However, these valid muscle coordination patterns are not arbitrarily different because, by construction, the geometric structure of the polytope that contains them defines strict spatial correlations among them (Kutch and Valero-Cuevas, 2012).

### System of linear equations to simulate static force production by a tendon-driven system

Consider producing a vector of static force with the endpoint of the limb in a given posture. The constraints that define that task (i.e., the direction and magnitude of the force vector at the endpoint) are linear equations (Valero-Cuevas, 2015) that come from the mapping between neural activation of individual muscles to static endpoint forces and torques the limb can produce. This mapping is linearly modeled by the equation

(1) $$\begin{array}{l} \left(\begin{array}{c} f_{x}\\ f_{y}\\ f_{z}\\ \tau_{x}\\ \tau_{y}\\ \tau_{z} \end{array}\right)=w=Ha=H\left(\begin{array}{c} a_{1}\\ a_{2}\\ a_{3}\\ ...\\ a_{n} \end{array}\right),a\in {\left[0,1\right]}^{n} \end{array}$$

where *H* is the matrix of linear constraints defined by the musculoskeletal anatomy of the limb (Valero-Cuevas et al., 2015b), **a** is the input vector of *n* muscle activations, and **f**∈ℝ^*m*^ is the m-dimensional limb output “wrench” (i.e., the forces and torques the finger can produce at the endpoint).

The output wrench, *w*, is at most 6-dimensional (i.e., 3 forces and 3 torques) depending on the number of kinematic degrees of freedom of the limb, and usually *m* < *n* because limbs have more muscles than kinematic degrees of freedom Valero-Cuevas (2015). Muscles can only pull, so elements of **a** cannot be negative, and are capped at 1 (i.e., 100% of maximal muscle activation).

What are the muscle coordination patterns that produce a given task? As explained in Valero-Cuevas (2015), the task of producing a static fingertip force vector is defined by specifying the desired values for the elements of the endpoint forces and torques of **w**. Each value yields a constraint equation, which in turn defines a hyperplane of dimension *n*−1, and their combination defines the task completely. The *feasible activation space* of the task, if it is well posed (Chvatal, 1983), is defined by the points **a** that lie within the *n*-cube and at the intersection of all constraint hyperplanes.

Geometrically speaking, the feasible activation space is a (*n*−*m*)-dimensional convex polytope *P* embedded in ℝ^*n*^ that contains all *n*-dimensional muscle coordination patterns (i.e., points **a**) that satisfy all constraints, and therefore can produce the task. Increasing task specificity by adding more constraints naturally decreases the dimensionality and changes the size and shape of the feasible activation space (Kuo and Zajac, 1993; Sohn et al., 2013; Inouye and Valero-Cuevas, 2016).

### The hit-and-run algorithm uniformly samples from feasible activation spaces

Calculating the geometric properties of convex polytopes in high dimensions is computationally challenging. Taking the generalized concept of an *n*-dimensional volume as an example of a geometric property of interest, the exact volume computations for n-dimensional polytopes is known to be tractable only in a polynomial amount of time (i.e., *#P*-hard) (Dyer et al., 1989). Currently available volume algorithms can only handle polytopes embedded in small dimensions like 10 or slightly more (Büeler et al., 2000). Studying vertebrate limbs in general, however, can require including several dozen muscles, such as our studies of a 17-muscle human arm and a 31-muscle cat hindlimb model (Valero-Cuevas et al., 2015b); and other models have over 40 muscles of the human lower limb (Arnold et al., 2010; Hamner et al., 2010; Kutch and Valero-Cuevas, 2012; De Sapio et al., 2014).

Similar difficulties arise when computing other geometric properties such as the shape and aspect ratio of *P* in high dimensions. We and others have described polytopes *P* by their bounding box (i.e., the range of values in every dimension) (Kutch and Valero-Cuevas, 2011; Sohn et al., 2013), but that singularly overestimates the shape and volume of the feasible activation space as discussed in Valero-Cuevas et al. (2015b). Consider a 3-muscle system with only one constraint, producing a 2-dimensional polygon as the feasible solution space. The bounding box of the polygon has a volume—even though a plane has zero volume—, and can be almost as large as the positive unit cube itself. Similar problems arise in the interpretation of the inscribed and circumscribed ball (Inouye et al., 2014).

We applied the Hit-and-Run method to sample points from the feasible activation space. We have presented a detailed explanation of the Theory (In Chapter 9 of Valero-Cuevas, 2015), and have justified the utility of this method on tendon-driven models of the index finger (Valero-Cuevas et al., 2015a). This complete probabilistic method describes the structure of feasible activation spaces *P* with a set of uniformly-at-random muscle activation patterns that produce the same wrench. This enables us to derive descriptive statistics, histograms, and point densities of the set of valid muscle activation patterns **a** uniformly sampled from the polytope. To do so, we use the Hit-and-Run method.

This approach can scale up to ~40 dimensions (i.e., limbs with ~40 independent muscles). This suffices to study extant vertebrate limbs, and thus compare, contrast, combine—and reconcile—today's three dominant approaches to neuromuscular control.

#### Example of a tendon-driven system

##### Realistic 3-D model of a 7-muscle human index finger

We applied this methodology to our published model of an index finger for static fingertip force production. The model is described in detail elsewhere (Valero-Cuevas et al., 2009a). Briefly, the input to the model is a 7-D muscle activation pattern **a**, and the output is a 4-D wrench **w** (i.e., static forces and torques) at the fingertip:

(2) $$\begin{array}{r} \text{w}=H\text{a} \end{array}$$

(3) $$\begin{array}{r} H=J^{-T}RF_{o},H\in {\mathbb{R}}^{4\times 7} \end{array}$$

where

(4) $$a=\left(\begin{array}{c} a_{FDP}\\ a_{FDS}\\ a_{EIP}\\ a_{EDC}\\ a_{LUM}\\ a_{DI}\\ a_{PI} \end{array}\right)$$

In Cartesian coordinates, the 4-D output wrench corresponds to the anatomical directions shown in Figure 1e.

(5) $$w=\left(\begin{array}{c} f_{x}\\ f_{y}\\ f_{z}\\ \tau_{x} \end{array}\right)=\left(\begin{array}{c} f_{radial}\\ f_{distal}\\ f_{palmar}\\ \tau_{radial} \end{array}\right)$$

The biomechanical model *H* includes three serial links articulated by four kinematic degrees of freedom (ad-abduction, flexion-extension at the metacarpophalangeal joint, and flexion-extension at the proximal and distal interphalangeal joints). The action of each of the seven muscles (FDP: *flexor digitorum profundus*, FDS: *flexor digitorum superficialis*, EIP: *extensor indicis proprius*, EDC: *extensor digitorum communis*, LUM: *lumbrical*, DI: *dorsal interosseous*, and PI: *palmar interosseous*) on each joint to produce torque is given by the moment arm matrix *R*∈ℝ^4 × 7^. Lastly, *J*∈ℝ^4 × 4^ and $F_{0}\in {\mathbb{R}}^{7\times 7}$ are the Jacobian of the fingertip with 4 kinematic degrees of freedom, and the diagonal matrix containing the maximal strengths of the seven muscles, respectively (Valero-Cuevas, 2000; Valero-Cuevas, 2015). The finger posture was defined to be 0° ad-abduction and 45° flexion at the metacarpophalangeal joint, and 45° and 10° flexion, respectively, at the proximal and distal interphalangeal joints.

##### Feasible activation space for a static fingertip force task

Our goal is to find the family of all feasible muscle activation patterns that can produce a given task. In particular, the task we explored is producing various magnitudes of a submaximal static force in the distal direction *f*_*distal*_ — in the absence of any τ_*radial*_, shown in Figure 1e. Therefore the feasible activation space is a polytope *P* in 7-dimensional activation space that meets the following *four* linear constraints in **a** (Valero-Cuevas et al., 1998; Valero-Cuevas, 2000; Valero-Cuevas, 2015)

(6) $$\begin{array}{r} f_{radial}=0 \end{array}$$

(7) $$\begin{array}{r} f_{distal}=\text{desired magnitude as}\%\text{of maximal} \end{array}$$

(8) $$\begin{array}{r} f_{palmar}=0 \end{array}$$

(9) $$\begin{array}{r} \tau_{palmar}=0 \end{array}$$

These four constraints on the static output of the finger yield a 3-dimensional (i.e., 7−4 = 3) polytope *P* embedded in 7-dimensional activation space. For details on how to create such models, apply task constraints and find such polytopes via vertex enumeration methods, (see Valero-Cuevas, 2015).

For the index finger model used in this paper, the published maximal feasible force in the distal direction is 28.81 Newtons. We defined the normalized desired distal task intensity as a value ranging between 0 and 1, i.e., each submaximal force can be produced by any of the points contained in its corresponding feasible activation space. For the production of a maximal force, the feasible activation space shrinks to a single point (Chao and An, 1978; Chvatal, 1983; Spoor, 1983; Valero-Cuevas, 2000).

#### Analysis of feasible activation spaces

##### Parallel coordinate visualization

**Figure 2:**
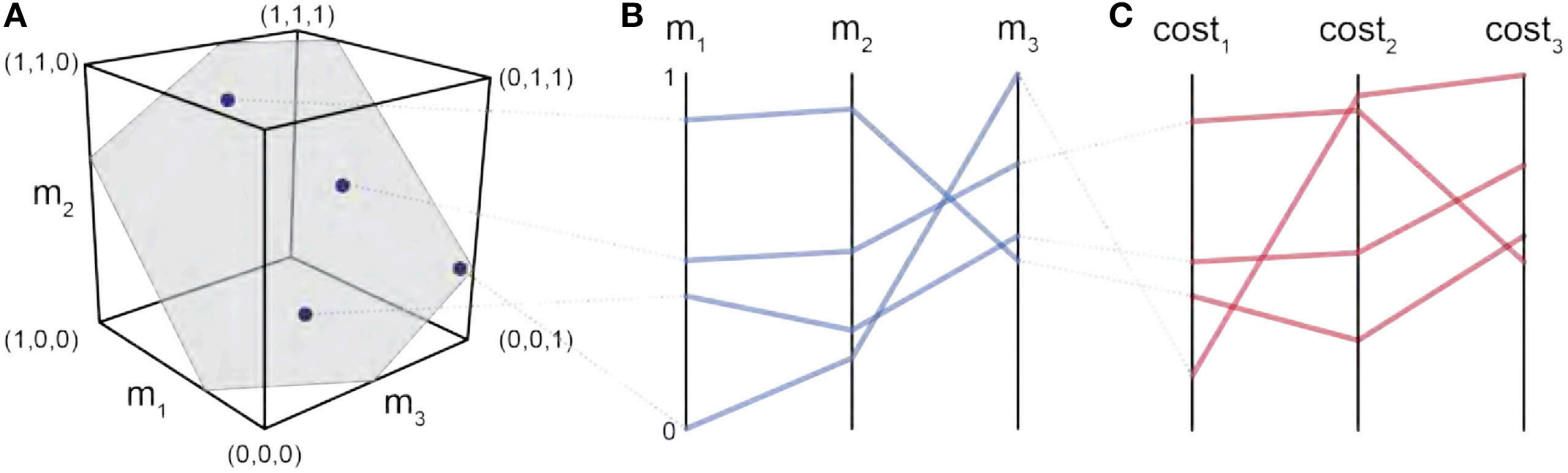
Parallel coordinates characterize the high-dimensional structure of a feasible activation spaces. Consider four points (i.e., muscle activation patterns) from the polygon that is a feasible activation space **(A)**. The activation level for each muscle (i.e., the coordinates of each point) are sewn across three vertical parallel axes **(B)**. As is common when evaluating muscle coordination patterns, each point can also be assigned a cost as per an assumed *cost function*. The associated cost for each muscle activation pattern can also be shown as an additional dimension. We show three representative cost functions **(C)**. Activation levels are bound between 0 and 1, and costs are normalized to their respective observed ranges.

For us to understand the structure of the feasible activation space, we aim to visualize the data. If we had a simple model with only three muscles (and one task force dimension), we could plot the feasible activation space as a plane within a 3D cube, as illustrated in Figure 2A. However, in our model, we have seven muscles. In our 3D reality, we cannot create a 7D scatter plot to highlight how muscle activation patterns are spatially located across the muscle dimensions, so we must project the data in a different way.

Parallel coordinates are a common graphical approach to visualize interactions among high-dimensional data (Krekel et al., 2010; Bachynskyi et al., 2013). To build familiarity with this visualization method, consider the results of a simple 3-dimensional (3-muscle) toy example shown in Figure 2A. This is the dimensionality of a finger with only 3 muscles, aiming to create a unidimensional pressing force. We begin by drawing *n* parallel vertical lines for each of the dimensions *n* (i.e., 3 muscles). With the axis limits of each line set between 0 and 1 (at the bottom and top of the plot, respectively), each muscle activation pattern (Figure 2A) is then represented by a zig-zag line that connects to the coordinates between 0 and 1 on each axis, as shown in Figure 2B. The blue zig-zag line that is connected at the top of *m*_1_ in Figure 2B represents the muscle activation point equal to (*m*_1_ = 0.8, *m*_2_ = 0.9, *m*_3_ = 0.4). You can see its corresponding location in the 3D cube, mapped to the parallel coordinate zig-zag line (the gray dotted line connects the two representations of the muscle activation pattern).

##### Neural and metabolic cost functions

As mentioned in the Introduction, the field of neuromuscular control has a long historical tradition of using optimization to find muscle activation patterns that minimize effort, which requires the (often contentious) definition of cost functions (Chao and An, 1978; Crowninshield and Brand, 1981; Spoor, 1983; Prilutsky, 2000). Therefore, we used four representative cost functions to calculate the relative fitness of each of the muscle activation patterns sampled—in effect also calculating the fitness landscape across all possible solutions. The cost functions are defined at the level of neural effort (*L*_1_, and *L*_2_ norms, representing the normalized sum of descending neural α-drive to the motor neuron pools); and at the level of metabolic cost, thought to be approximated by neural drive weighted by the strength of each muscle ($L_{1}^{w}$ and $L_{2}^{w}$ norms) (Crowninshield and Brand, 1981; Prilutsky, 2000).

To visualize the costs associated with each valid muscle coordination pattern we simply added three vertical lines at the far right of the parallel coordinates plot, one for each of the three cost functions, as shown in Figure 2C. The variables *a*_*i*_ and *F*_0*i*_ represent the activation of the *ith* muscle in a given muscle activation pattern, and the maximal strength of each muscle (Crowninshield and Brand, 1981; Prilutsky, 2000). Maximal muscle strengths are approximated by the multiplying each muscle's physiological cross-sectional area, in *cm*^2^, by the maximal active muscle stress of mammalian muscle, 35*N*/*cm*^2^ (Zajac, 1993). These four cost functions are but four examples from the literature; an investigator is free to use this visualization of the feasible activation space with any cost function deemed relevant to their study.

##### Histograms of the activation level of each muscle across all valid solutions

Muscle-by-muscle histograms are another straightforward way to visualize the many points sampled from the convex polytope. Histograms are particularly helpful because they illustrate the structure of the space of all feasible activations, allowing us to see which muscle activation patterns are on the edge of the space, which solutions exist in the middle of the space, and how the bounds of the space and the distribution change across different tasks (in this case, as the task force increases). They visualize the relative number of solutions (i.e., density of solutions) that required a particular level of activation from a particular muscle within its range of [0, 1]. In addition, the upper and lower bounds of the histograms show, in fact, the size of the side of the bounding box of the polytope in every dimension (i.e., for each independently controlled muscle).

##### Dimensionality reduction

Investigators have repeatedly reported that electromyographical signals (i.e, experimental estimates of muscle activation patterns) tend to exhibit strong correlations with one another. In these experimental descriptions of dimensionality reduction of neuromuscular control only few independent functions—sometimes called synergies—suffice to explain the majority of the variability in the observed muscle activation patterns (Krishnamoorthy et al., 2003; Dingwell et al., 2010; Kutch and Valero-Cuevas, 2012; Alessandro et al., 2013; Bizzi and Cheung, 2013; Steele et al., 2013, 2015). Principal components analysis (PCA) is a widely used technique to extract these few independent basis functions (correlation vectors called principal components, PCs) from high-dimensional data (Clewley et al., 2008). In this case, PCs are often called the experimental representations of synergies of neural origin (Kutch and Valero-Cuevas, 2012).

Therefore, we applied PCA to points (i.e., muscle coordination patterns) sampled from the feasible activation space at each force level. This provides the PCs that describe the correlations among valid muscle activation patterns for a given task. For example, the feasible activation space *P* in a 3-muscle system with one constraint is a 2-dimensional polygon embedded in 3-dimensional activation space. Thus, applying PCA to points sampled from the polygon will extract 2 synergies (i.e., 3-dimensional correlation vectors PC1 and PC2) that wholly explain the feasible activation space. By extension, in the case of fingertip force production in Figure 1, the feasible activation space is a 3-dimensional polytope embedded in the 7-dimensional activation space. PCA should also extract, by construction, as many synergies as there are dimensions in the feasible activation space. For static force production with the index fingertip (i.e., 7 muscles and 4 constraints), we know that 3 principal components will describe 100% of the variance in points sampled from the feasible activation space (i.e., 7-dimensional correlation vectors PC1, PC2, and PC3).

Applying PCA to our data allows us to test whether and how its results change when applied to feasible activation spaces for different magnitudes of fingertip force. We applied PCA to feasible activation spaces for fingertip task intensities ranging from 0 to 90% of maximal. Specifically, we applied the *prcomp* function in R, and specified that the calculation operates on the covariance matrix of the raw data. We compare both the variance explained by each PC and their loadings (e.g., correlations among muscles) as the force level increases (Valero-Cuevas et al., 2016). Lastly, we tested whether the dispersion (i.e. the two central quartiles) and median of our PCA estimates are sensitive to the number of points sampled from each feasible activation space. This is important in practice because experimental studies tend to record and analyze a practical number (e.g., 10) of repetitions of the same motor task from a given subject, and aggregate data from different subjects (Valero-Cuevas and Santello, 2017). Although we have reported that subjects tend to exhibit similar muscle activations for a given task (Valero-Cuevas, 2000), performing dimensionality reduction on such few trials and across multiple non-identical subjects (i.e., samples in Figure **5**) may lead to imprecise (i.e., uncertain) estimates of the synergies when sampling from high-dimensional spaces.

## 3. Results

We used our realistic index finger model to calculate the feasible activation space for the task of producing static fingertip force in the distal direction (see Figure 1). By showing how this same space can be interpreted from three dominant perspectives, we propose a conceptual paradigm to unify today's theories of neuromuscular control. The model contains the contribution of each of the seven muscles of the finger to the resultant static fingertip force vector (Valero-Cuevas, 2015). As described briefly in the Methods, all valid muscle activation patterns to produce a given fingertip force vector (i.e., all ways in which one can combine the actions of the seven muscles to produce a given fingertip force vector) are contained in a low-dimensional polytope embedded in 7-dimensional space. Hit-and-Run is a method for uniform polytope sampling that collects thousands of muscle activation patterns, which become a valid geometric approximation to the structure of the feasible activation space (Valero-Cuevas et al., 2015a). We examined how these feasible activation spaces (and their alternative representations) change with increasing task intensity (i.e., fingertip force magnitude, Figure 1e). In particular, we studied task intensities between 0% (i.e., pure co-contraction without output force) and 100% of maximal static force (i.e., a unique solution Valero-Cuevas et al., 1998).

### Parallel coordinate visualization naturally reveals the structure of the feasible activation space

**Figure 3:**
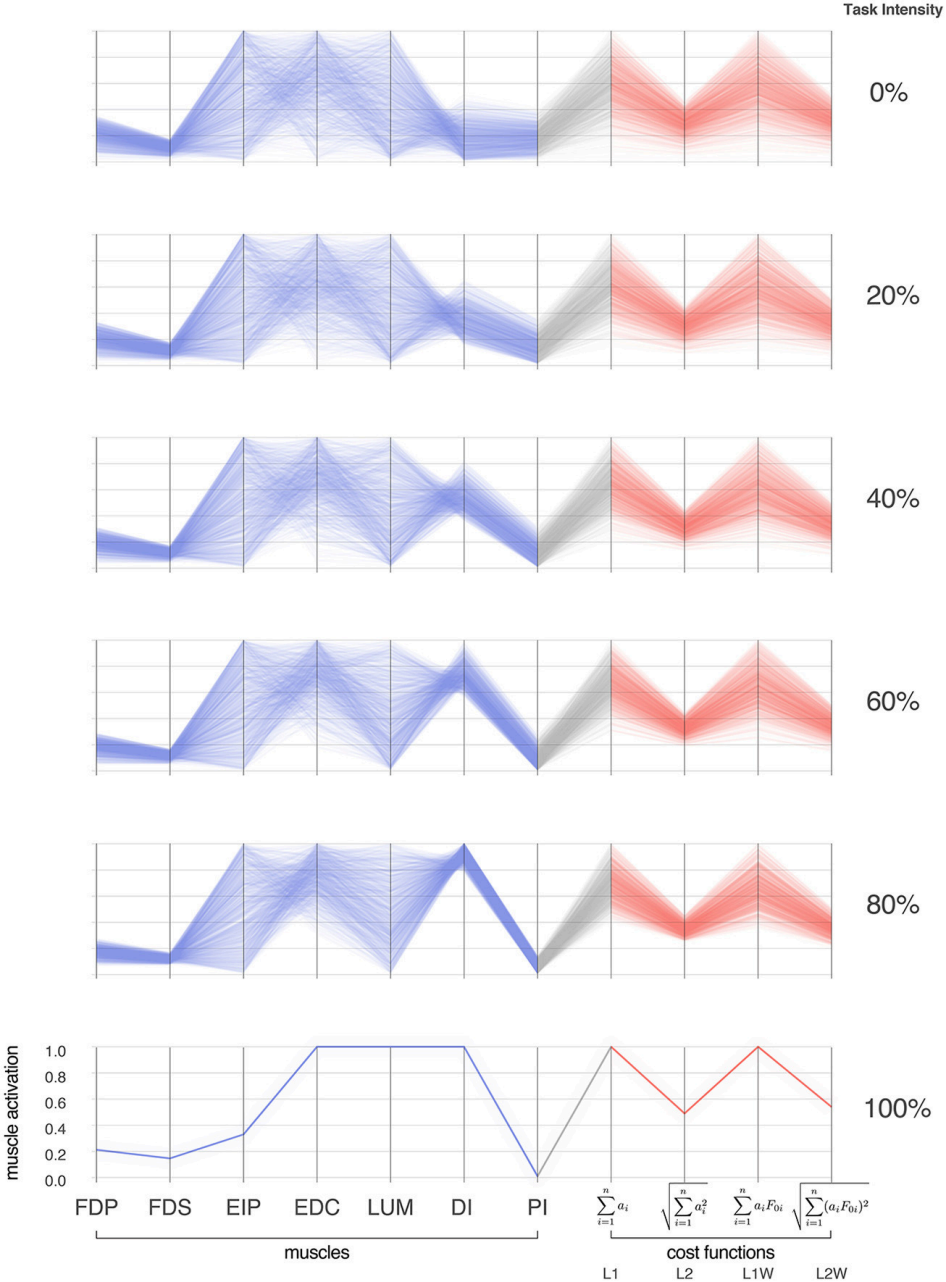
Activation patterns of the seven muscles of the index finger across six intensities (magnitudes) of a fingertip force vector in the distal direction. The connectivity across parallel coordinates visualizes the correlations among muscle activation patterns at different task intensities. At the extremes of 0 and 100% we have, respectively, the coordination patterns that produce pure co-contraction and no fingertip force, and the one unique solution for maximal fingertip force (Valero-Cuevas et al., 1998). In between, we see how the structure of the feasible activation spaces changes, and that much redundancy is lost rather late (at intensities >80%, in agreement with Sohn et al., 2013). In blue are the activation values, and in red are normalized costs for four common cost functions in the literature. For each task intensity, we produced 1,000 points that are uniformly distributed in the polytope via the Hit-and-Run method. The muscles are *FDP: flexor digitorum profundus, FDS: flexor digitorum superficialis, EIP: extensor indicis proprius, EDC: extensor digitorum communis, LUM: lumbrical, DI: dorsal interosseous, PI: palmar interosseous*. Color is used solely to differentiate muscle activations (blue) from cost values (red).

Parallel coordinate visualization effectively reveals correlations that exist among the 1,000 valid muscle activation patterns for each intensity of desired fingertip force, and activation pattern cost, Figures 2, 3.

**Figure 4:**
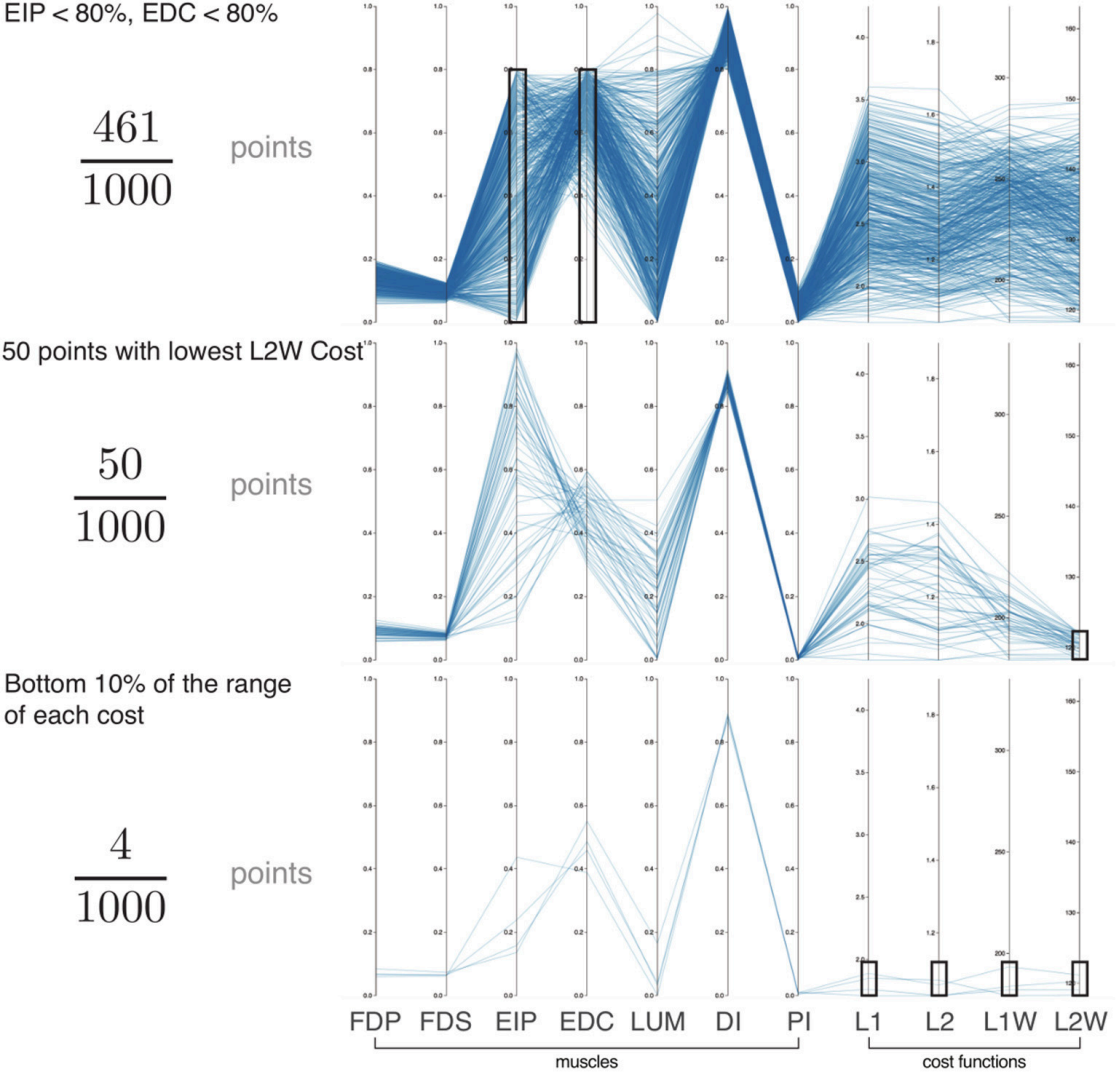
Exploration of the feasible activation space for task intensity of 80%. Here we show three informative examples of constraints applied to the points sampled from the feasible activation space (*n* = 1,000; axes match those of Figure 3). With this interactive visualization, we can easily see how the size (i.e., number of solutions) and characteristics of the family of valid muscle activation patterns change. For example, in the event of **(Top)** weakness of a group of muscles (54% reduction), **(Middle)** selection of the lowest 5% of a given cost function (95% reduction), and **(Bottom)** enforcing the lowest 10% of cost range across multiple cost functions (99.6% reduction). In all cases, the family of valid muscle activation patterns retains a wide range of activation levels for some muscles. While it is challenging to understand the structure of the feasible activation space with a static plot of the parallel coordinates, interactively manipulating the muscle ranges on one or multiple axes makes it very easy to view and describe how muscle activations change in the face of different constraints.

Parallel coordinate visualization provides deep insight into the interactions among muscles that can produce a given task. Because it allows interactive exploration of the feasible activation space, one can restrict the activation level of any one or multiple muscles to see the associated activation levels of the remaining muscles (i.e., see a subsample of the feasible activation set). Figure 4 shows how, for 80% of task intensity, only 46% (i.e., $\frac{461}{1,000}$) of all possible solutions survive when we only keep solutions where EIP and EDC are below 80% of maximal excitation. We chose to limit the extensors, as they are both innervated by the radial nerve and are susceptible to limitation from, for example, neuropathy or stroke. This robustness-related system behavior is visible in other muscle pairs via the interactive parallel coordinates plot. We find that even a minor neural or muscle dysfunction can disproportionally compromise the solution space—even for sub-maximal forces. These results further challenge the definition of muscle redundancy as discussed in detail in Kutch and Valero-Cuevas (2011), Valero-Cuevas (2015), Marjaninejad and Valero-Cuevas (2019), in that our description of redundancy may need to incorporate the structure of the feasible activation space to best describe how motor control can occur with perturbation to one or more muscles.

While we know from experience that a limitation on one muscle yields compensation from the others, Figure 4 explains why, and how much to expect. All data used for Figure 4 are for a task intensity of 80%. When we select only the lowest 5% of L2 weighted costs (Figure 4, middle figure) there exist many “near-optimal” solutions that are dramatically different (note the broad ranges and criss-cross patterns in the second panel of in Figure 4). This wide space exists in spite of this strong criterion.

Evaluating the slope of the lines connecting muscles enables an intuitive understanding of inter-muscle correlations. The Pearson product-moment correlation coefficients were 0.99, −0.50, and −0.06 in the adjacent muscle pairs FDP—FDS, LUM—DI, and EIP—EDC, respectively. The interactive parallel coordinate visualization also allows for any pairwise comparison by simply dragging and reordering the vertical axes. This is an effective *ad-hoc* method to viewing the inter-muscle correlations for exploratory data analysis.

### Low-dimensional approximations to the feasible activation space

We applied Principal Component Analysis (PCA) to sampled muscle activation patterns for 10 levels of task intensity. However, to replicate the fact that experimental studies can only collect a finite amount of data from each subject, we did this in an iterative fashion as follows. We collected 10,000 points sampled uniformly at random from each feasible activation space via Hit-and-Run (Valero-Cuevas et al., 2015a). From these 10,000 points, we sampled 10, 100, and 1,000 points at random (to simulate “experimental” sample sizes), and applied PCA to each set of sampled points. For each of the sample sizes, we replicated the sampling 100 times, producing a distribution of principal component results, and thus, a distribution of variance-explained metrics for PC1 (and the same for the other components). This bootstrap analysis serves to inform how many samples one must collect from a subject to get an effective set of principal components. The *H* matrix was fixed across all replicates and samples.

**Figure 5:**
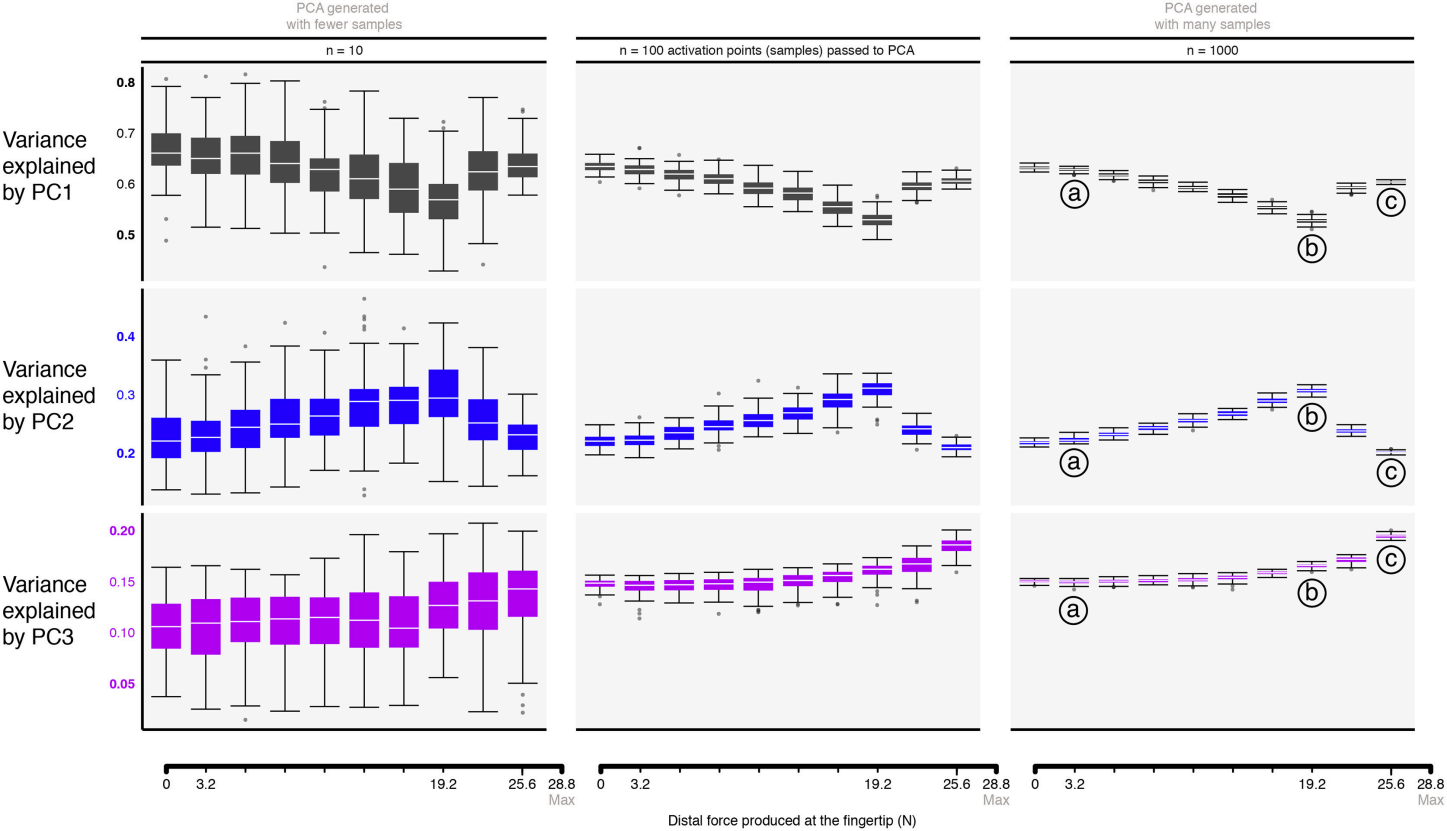
Approximating the structure of feasible activation spaces via principal components analysis (PCA) is sensitive to both the task intensity and the amount of input data used. Rows show the variance explained by the first (top) through third (bottom) principal components with increasing data points for a given replicate (left to right). Hit-and-Run sampling provides the ground truth for the high-dimensional structure of the feasible activation set at each task intensity. Each box plot, across all subplots, is formed from 100 metrics (replicates), where each metric is the PC variance explained for a replicate “subject” which performed the task *n* times (where n is one of 10, 100, or 1000 task repetitions). We find that PCA approximations to this structure do not generalize across tasks intensities (i.e., the polytope changes shape as redundancy is lost), and numbers of points. That is, > 100 muscle activation patterns should be collected from a given subject to confidently estimate the real changes in variance explained as a function of task intensity. Compare points labeled a, b, c, corresponding to 11, 66, and 88% of task intensity, respectively.

**Figure 6:**
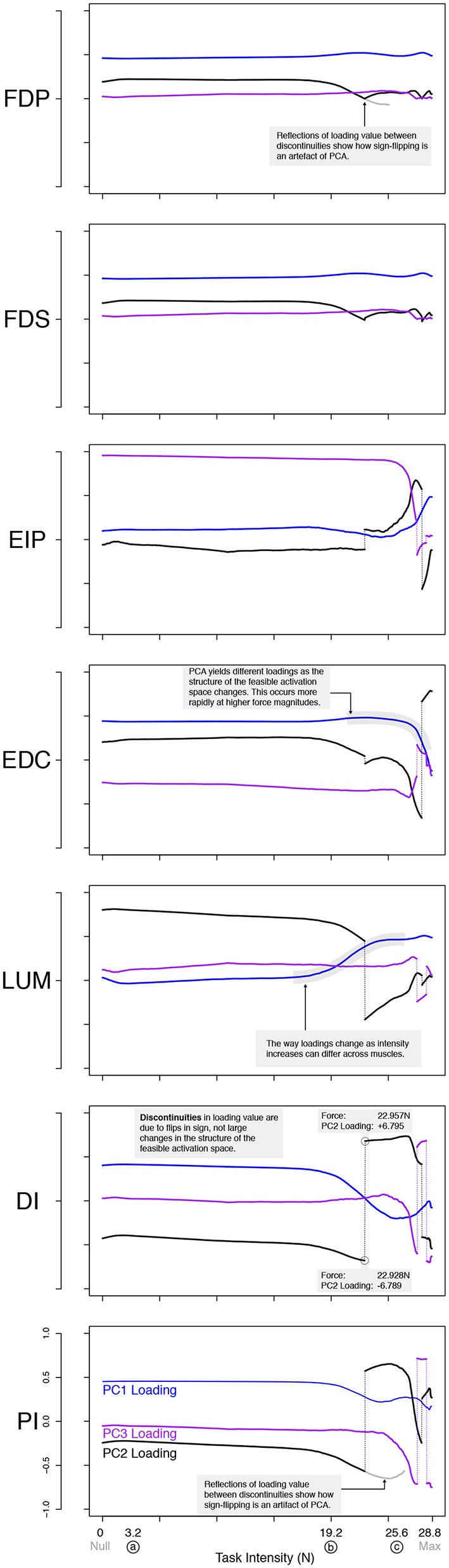
PCA loadings change with task intensity. For each of 1,000 task intensities, we collected 1,000 muscle activation patterns from the feasible activation space and performed PCA. The facet rows show the changes in PC loadings, which determine the direction of all PCs in 7-dimensional space. Note that the signs of the loadings depend on the numerics of the PCA algorithm, and are subject to arbitrary flips in sign (Clewley et al., 2008)—thus for clarity we plot them such that FDP's loadings in PC1 are positive at all task intensities. Dotted vertical lines connect loadings of PC2 and PC3 in spite of flips in sign. A discontinuity here is not indicative of a major change to the feasible activation space. It instead, is a result of how PCA selects loadings. The shape of the activation space has tilted at these points, thereby flipping the sign. Note that the values are the same before and after the jump, less the sign. These loadings (i.e., synergies) change systematically, as noted for representative task intensities a, b, c in Figure 5, and more so after b. This reflects changes in the geometric structure of the feasible activation space as redundancy is lost.

Figure 5 shows the box plots describing the variances explained by the three principal components (PC1, PC2, and PC3) across task intensities. The third PC, PC3, explains the remainder of the variance (13—15%) for the resulting 3-dimensional polytope. Recall that the 4 task constraints (*f*_*radial*_, *f*_*distal*_, *f*_*palmar*_, τ_*palmar*_) applied to 7 muscles yield a 3-dimensional polytope embedded in the 7-dimensional muscle activation space (Valero-Cuevas et al., 1998); as such, the sum of all three PCs is exactly 100%. The supplemental website (linked in the Data Availability Statement below) contains alternate versions of Figure 6 with varying input transformations.

The box plots in Figure 5 quantify how different amounts of data change the estimates of variance explained by a PC with task intensity (c.f. labels a vs. b vs. c). We see this dispersion is small in the center and right columns. Note that the ratio of variance explained between PC1 and PC2 between 50 to 80% of task intensity reveals changes in the aspect ratio of the feasible activation space with task intensity.

Importantly, we observe how using experimentally realistic sample sizes of 10 same-task repetitions per subject (the leftmost column in Figure 5) not only does not capture this change, but its standard deviation is large enough to blur the notable differences that are known to appear with larger (but experimentally unrealistic) sample sizes. The impact of impoverishing the number of independent samples fed to PCA reminds us that inadequate amounts of data obfuscate the underlying changes in the structure of the data analyzed (Figure 5).

There were also changes in the loadings of the PCs, especially above 60% task intensity. While the ratio of variance explained between PC1 and PC2 gives a sense of the aspect ratio of the feasible activation space, the loadings of PC1 and PC2 speak to its orientation (Valero-Cuevas, 2015; Valero-Cuevas et al., 2016). Figure 6 shows how the loadings of PC vectors change across labels a, b, and c, Figure 5. These loadings indicate that the orientation of the feasible activation space in 7-dimensional space changes mildly at forces <65% of the maximal task force, and changes more dramatically with higher forces.

These changes we see in (i) the lower and upper bounds of activations, (ii) the relative variance explained and (iii) the loadings for all three PCs, demonstrate that the size, shape, and orientation of the feasible activation space changes with task intensity. The muscle activation distribution “between the bounds” has profound implications for prior work which chiefly examines the ultimate upper- and lower-bounds of activation for tasks in different directions (Simpson et al., 2015; Valero-Cuevas et al., 2015b). Moreover, detecting changes in these high-dimensional structures is done in the best-case scenario, as it exists in the absence of experimental noise, within- and across-subject variability, and measurement error. As will be elaborated in the Discussion, this implies that PCs (i.e., synergies) are laborious to obtain experimentally, and even then do not necessarily generalize across intensity levels.

### Changes in the probabilistic structure of the feasible activation space with increasing task intensity, or how muscle redundancy is lost

**Figure 7:**
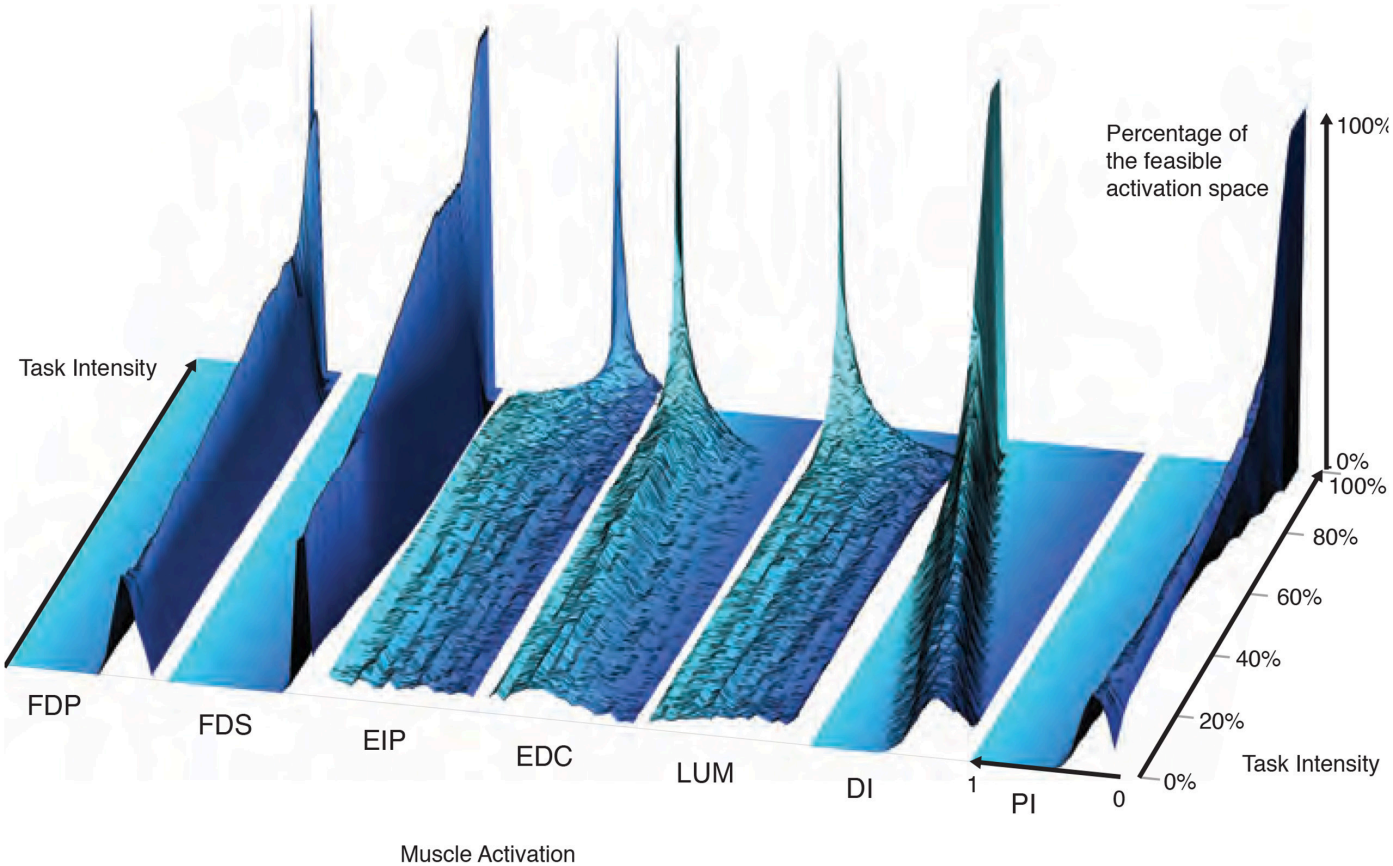
The within-muscle probabilistic structure of feasible muscle activation across 1,000 levels of fingertip force intensity. The cross-section of each density plot is the 50-bin histogram of activation for each muscle, at that task intensity. The changes in the breadth and height for each muscle's histogram reveal muscle-specific changes in their probability distributions with task intensity. Height represents the percentage of solutions for that task. The axis going into the page indicates increasing fingertip force intensity up to 100% of maximal. Color is used to provide perspective. It is interesting to note that, for example, both extensor and flexor muscles are used to produce this “precision pinch” force. This is to be expected as the activity in the extensors is necessary to properly direct the fingertip force vector (Valero-Cuevas and Hentz, 2002).

The maximal static fingertip force vector in a given direction is produced by a single and unique combination of muscle activations. In contrast, any sub-maximal magnitude of that same vector is produced by an infinite number of solutions (Chao and An, 1978; Spoor, 1983; Valero-Cuevas, 2000; Valero-Cuevas, 2015). Our analysis of feasible activation spaces at different task intensities also allows us to characterize how this redundancy changes, and is eventually lost. The histogram heatmaps in Figure 7 illustrate the changes and shrinking of within-muscle histograms (the space upon which probability density functions must operate) of valid activation levels across task intensities, converging to a single solution at maximal force output. These surface plots show how the normalized histograms (of 1,000 valid activation levels for each muscle at each intensity level) change at each of 100 equally-spaced levels of task intensity between 0 and 1. Following a muscle's column from bottom to top shows the activation histograms converge, naturally, to a spike at the unique value for maximal force production.

The low flat areas on the sides of each surface plot (e.g., clearly visible for DI) represent muscle activation levels that are not valid for that task intensity. That is, there exist no valid muscle activation patterns that contain that muscle at that level, and thus no points are found there.

These plots show *within-muscle probability functions* and the rate of convergence to the unique solution for maximal force output across muscles. This is in contrast with the parallel coordinate plots in Figure 3 that shows the *correlation across muscles*. Importantly, the histograms of activation levels for each muscle need not be symmetric, nor have the same shape (skewness and kurtosis) as the magnitude of the output force increases. For some muscles, the convergence accelerates after 60 or 80% of task intensity (as in LUM and EIP), while others converge monotonically along the entire progression (e.g., DI and PI). The peaks (i.e., modes or most common values) of each histogram at each task intensity represents the slice of the polytope that has the largest relative volume along that muscle's dimension (i.e., greatest frequency of that level of muscle activation across all valid solutions). Importantly, for most muscles (FDP, FDS, EIP, EDC, and LUM), the mode is not necessarily located at the same relative level of activation needed for maximal force output—even when scaling it linearly with task intensity. That is, the histogram at high levels of force is not simply a shifted version of the histogram at low levels of force. The histograms for DI are the exception, whose modes seem to scale linearly with task intensity.

These histograms and the parallel coordinate visualizations demonstrate that the probabilistic and correlation structure, respectively, of feasible activation spaces, do not necessarily generalize across task intensities. Nor can they be inferred from their bounding boxes alone (i.e., upper and lower activation bounds for each muscle). An immediate example is how, for most task intensities, both EIP and LUM have similar lower and upper bounds near 0 and 1, respectively—yet their distributions are thoroughly distinct.

## 4. Discussion

### Summary

Feasibility Theory, as a conceptual and computational approach, is a means to pierce the curse of dimensionality to establish a physics-based ground truth for neuromuscular control. This practical approach can now characterize—in an arguably complete way—the space of all valid ways to activate multiple muscles to produce a given task. This initial presentation is limited to the case of static force production. Additional work is needed to extend to sequences of tasks, as has been done for optimization during gait analysis—where the dynamical constraints during movement are applied in the context of static optimization (Anderson and Pandy, 2001; Simpson et al., 2015). But we can already say that feasible activation spaces are, in fact, *the* high-dimensional landscapes upon which all neuromuscular learning, control, and performance must occur. These landscapes are predicated upon the strong experimental evidence for linearity in tension-to-force transduction in cadaveric (Kutch and Valero-Cuevas, 2011), live (Kamper et al., 2006), and modeled (Synek and Pahr, 2016) studies. Therefore, they provide an integrative and unifying perspective that demonstrates how today's dominant theories of neuromuscular control are alternative approximations to feasible activation spaces from optimization, synergistic, and probabilistic perspectives. Feasibility Theory unifies these alternative approaches to motor control in the sense that feasible activation spaces represent an objective conceptual and computational common ground for these theories.

Changes in the structure of the feasible activation space do not imply a given control strategy. They merely establish the bounds within which a species evolves a control policy for a given body morphology. It is possible that the nervous system operates within a very small subset of this space—which could be described by different principal components and even probability distribution functions. Feasibility Theory, however, allows us to formally phrase and test such hypotheses.

### The value of a cost function

Optimization is the oldest computational approach to finding valid muscle activation patterns that produce limb function (e.g., Chao and An, 1978). While optimization is, of course, a reasonable hypothesis to explore neuromuscular control (Todorov and Jordan, 2002), some criticize it as a mathematical abstraction that anthropomorphizes neurons with the ability to choose, evaluate and follow cost functions in high-dimensions (De Rugy et al., 2012; Loeb, 2012). There is, nevertheless, an intimate relationship between optimization and feasible activation spaces (Chvatal, 1983). Optimization is analogous to finding the best solution in the dark—guided by repeated small steps based on evaluations of cost- and constraint-function. Computing the feasible activation space is then a means to “turn on the lights” to see all possible valid solutions independently of cost (Valero-Cuevas, 2015). Our complete sampling of high-dimensional feasible activation spaces (Smith, 1984; Lovász, 1999) allows us to compare and contrast *families* of solutions as per alternative cost functions instead of *individual* optimal solutions for a particular cost function. Figure 3 demonstrates a complete description of families of valid coordination patterns and their relationship to alternative cost functions. Importantly, similar valid muscle activation patterns can have dissimilar costs and vice versa.

Thus, Feasibility Theory allows us to compare, in detail, alternative “cost landscapes” across the entire set of feasible motor commands. By not having to insist on (or settle for) individual optimal—or near-optimal—solutions, we now have the same ability the nervous system has to explore, compare, and contrast multiple valid (be they optimal or suboptimal) ways to coordinate muscles. Importantly, the relationships among valid muscle activation patterns emerge naturally from the physical properties of the limb and definition of the task. This *cost-agnostic* approach allows us to re-evaluate our assumptions about what the nervous system cares—and does not care—about. Lastly, this cost-agnostic approach also provides a powerful tool for inverse optimization, i.e., uncovering latent cost functions from data (Tsirakos et al., 1997). Our comparison across cost functions using parallel coordinates is already a form of inverse optimization.

### Freedom under constraints

We have so far only used “hard” task constraints which must be met exactly. However, Feasibility Theory also holds for soft constraints. For example, if a tendon-driven system is required to produce a 3D force vector in general distal direction and of a general magnitude (defined, say, as a sphere of 1.0 N radius centered on the nominal force), then we can apply these tolerances to the constraints defining the task. In effect, Feasibility Theory allows us to study both soft and hard constraints where the latitude of the accuracy of the task naturally defines the precision with which muscle activation patterns must be selected. One can define the task intensity to be, say, anywhere between 50 and 60%, and study the concomitant increase in options available to produce forces within that range. Thus, one can characterize the changes in the feasible activation space as the task constraints are relaxed or tightened. Similarly, adding task constraints, such as the need to produce a particular stiffness at the endpoint (Inouye and Valero-Cuevas, 2016), naturally reduces the dimensionality of the feasible activation space.

### How to apply feasibility theory in an experiment

The most important input to this analysis is the relationship between muscles and the endpoint wrench. With this relationship composed as the *H* matrix as in 1, and a desired wrench *w*, Hit-and-Run can be used to produce parallel coordinate plots and density histograms for static force production with vertebrate limbs. For example, using a measure of muscle activation (such as fine-wire EMG), an experimentalist can compare the muscle activation pattern chosen by a research participant in comparison to the full feasible activation space that could achieve the same force, and see how those patterns change across fatigue, disability of a muscle, or manipulation of the feedback. After a tendon-transfer surgery, for example, the subject may initially inhabit only a specific part of the feasible activation space to produce a task, but must use feedback from the parallel coordinate plot to find solutions which take less effort. In effect, visualizing the entire feasible activation space could help us understand how rehabilitation can be guided toward more advantageous local minima (Towles et al., 2008).

In parallel, a scientist with a cost function to test on a model can quickly identify how different cost function parameters can affect the space of feasible activations, and see how specific the global optima is, with respect to other muscle activation patterns. Importantly, anthropometric differences affect the shape of the feasible activation space, so those subject-specific differences must be either incorporated or may be addressed through sensitivity analysis (such as Monte-Carlo manipulation of moment arm values, as in Valero-Cuevas et al., 2015b).

### Extension to dynamical force production or movement

Limbs are valuable for more than just their ability to produce isometric forces. First, there is the extension to “non-static isometric” force production (e.g., rotating a grasped object with respect to gravity), which must contend with time-varying muscle activation-contraction dynamics and target grasp wrench (i.e., such that the object is always securely held against a time-varying gravity vector Rácz et al., 2012). Joint angles, the end-effector Jacobian, moment arm matrix, and vector of maximal feasible contraction levels per muscle will vary nonlinearly, and with kinematic redundancy as a possibility for a given endpoint location, we can introduce multiple feasible activation spaces that are capable of producing a given task force. Even a simple task in the workspace likely exhibits redundancy at different levels of abstraction, where redundancy is sourced from feasible activation spaces and joint null spaces simultaneously.

As muscles exhibit state dependence, the ability of an animal to produce precise dynamic forces is affected by the tendon tensions from moment to moment. The inter-muscle dynamics across a human index finger, for example, would necessarily require a feasible activation trajectory—which may or may not be representable by a convex hull. Applying Feasibility Theory to non-static isometric force production may require detailed investigation into the dynamics of musculoskeletal force transduction. In parallel to the dynamics, non-convexities may emerge from neural constraints or even nonlinearities and hysteresis of muscle function.

**Figure 8:**
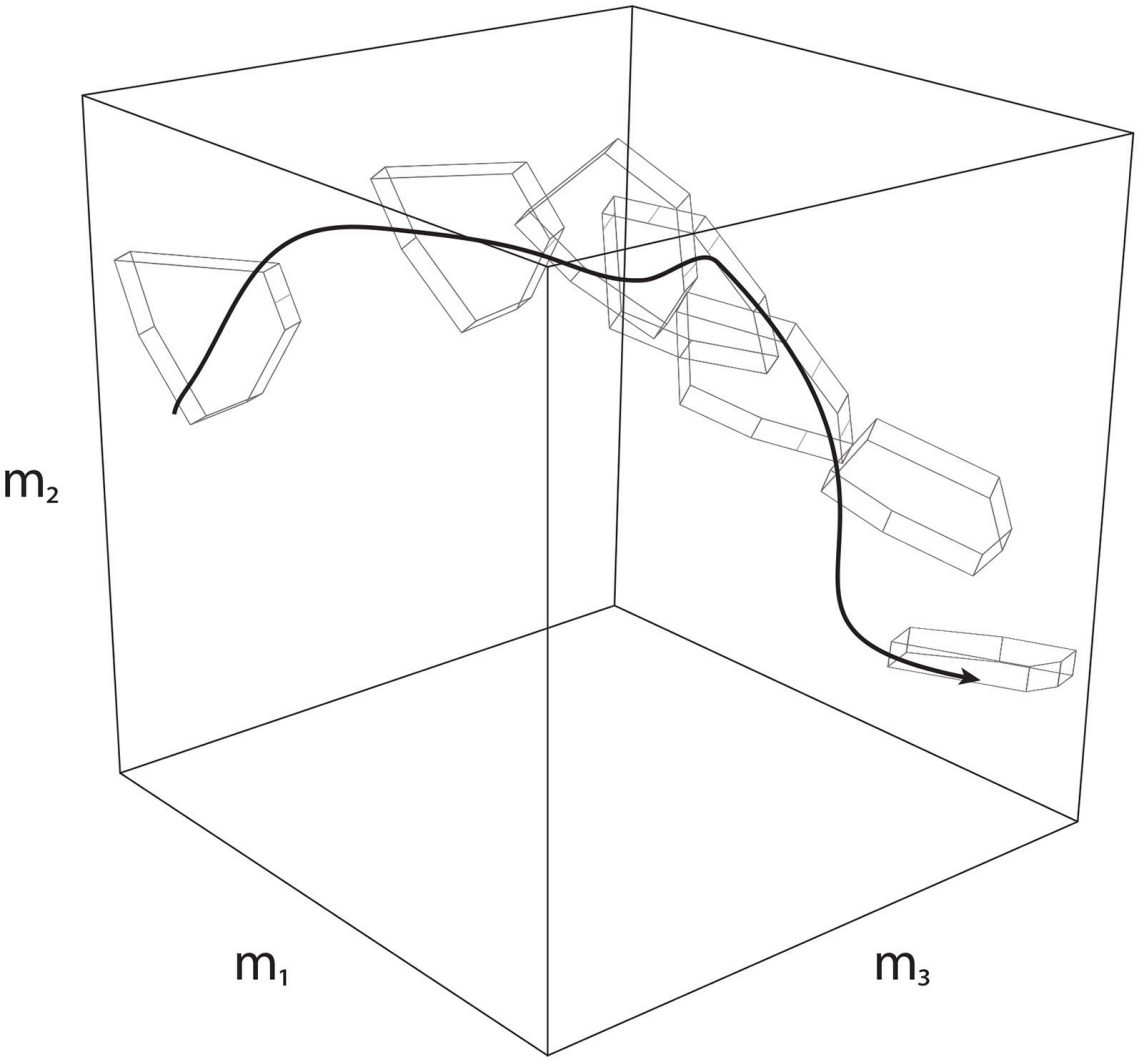
Spatiotemporal Tunneling. A dynamical movement can be decomposed into a sequence of slices in time, where each slice has a corresponding feasible activation space. Strung together, the sequence of feasible activation spaces form the “spatiotemporal tunnel” through which the neuromuscular system must operate. In this 3-dimensional schematic example, the black line represents one valid time-varying sequence of activations for three muscles. Because this sequence exists within each feasible activation space, it necessarily meets the constraints of the dynamical task at each instant.

Secondly, Feasibility Theory can be extended to address dynamical behavior by applying it to a sequence of slices in time. That is, a dynamical task can be equivalently analyzed as a sequence of “slices” (Anderson and Pandy, 2001; Cianchetti and Valero-Cuevas, 2009; Simpson et al., 2015; Trinler et al., 2018)—where one can define a feasible activation space at each slice to determine how the nervous system must change activation patterns such that it is always implementing a valid solution (Simpson et al., 2015). When strung together, these individual spaces give rise to a “spatiotemporal tunnel”—the time-varying extension of the feasible activation space (Figure 8).

### Structure, correlation, and synergies

The physical properties of the limb and the definition of the task together give rise to a low-dimensional structure of the feasible activation space (Valero-Cuevas, 2015). Therefore, experimental recordings of muscle activations during limb function will exhibit a dimensionality that is smaller than the number of muscles (Tresch and Jarc, 2009; Kutch and Valero-Cuevas, 2012; Alessandro et al., 2013). Thus, applying PCA to the points sampled from the feasible activation space will inevitably find that few PCs can explain the variance in the data (Brock and Valero-Cuevas, 2016).

Our application of PCA at increasing task intensities (i.e., as muscle redundancy is lost) allows us to demonstrate—for the first time to our knowledge—several important features and limitations of dimensionality reduction. For example, we see that the aspect ratio (Figure 5) and orientation (Figure 6) of the feasible activation spaces change as their size shrinks (Figure 7). Thus, such *descriptive* synergies (Brock and Valero-Cuevas, 2016) extracted from limited experimental observations likely do not generalize well across task intensities. Producing further insights into the feasibility-synergy relationship necessitates more objective metrics of the feasible activation space's structure.

The intensity-dependent structure of feasible activation spaces also has important consequences for motor control and learning. Producing force vectors at the endpoint of a finger or limb with accurate magnitude and direction are critical for versatile manipulation and locomotion (Valero-Cuevas et al., 1998; Donelan et al., 2004; Cole, 2006). If a given synergy can produce such accurate force vectors only for a given task intensity (and thus inaccurate vectors at other intensities), then the attractiveness of task-specific synergies to simplify the neuromuscular control of the limb is reduced. Although we do not present an analysis of task-irrelevant synergies, data from this paper can be concatenated prior to PCA analysis to explore how principal components vary across the entire distal task.

To compensate, the nervous system would need to learn, recall, and implement intensity-specific synergies. Prior experimental work has shown that the nervous system produces accurate fingertip forces of different magnitudes by, instead, likely scaling a remembered muscle activation pattern to produce forces of different magnitudes (Valero-Cuevas, 2000), together with full-dimensional error correction (Valero-Cuevas et al., 2009b). The observation of higher forces yielding more variable PC loadings indicates that lower dimensional substructures could approximate low- and medium-level forces for a given direction, motivating further analyses of PCA effectiveness across task-intensity (and with NMF, for example).

Our results also show how experiments with realistically moderate numbers of participants and test trials likely do not contain sufficient information to produce robust estimates of descriptive synergies across task intensities. As per the curse of dimensionality, sampling uniformly at random from high-dimensional spaces is exponentially difficult. Thus, even for this anatomically complete 7-muscle finger model, PCA depends strongly on the number of independent observations, such as uncorrelated trials from one subject or different subjects. Figure 5 shows that 100 to 1,000 such ideal data points from a simulated “test subject” are needed to produce accurate estimates of changes in the PCs with task intensity (c.f. labels a vs. b vs. c). Future studies should explore how many experimental data points are sufficient from a given subject when recording from only a subset of the many (20+) muscles of human limbs in the presence of experimental noise, inherent stochasticity of EMG, and within- and between-subject variability. Some studies have begun to ask subjects to explore different ways to perform a given task (Kuxhaus et al., 2005; Berger and d'Avella, 2014) (i.e., estimate the structure of the feasible activation space), but in practice, such studies cannot likely collect sufficient data uniformly at random to obtain accurate estimates of the descriptive synergies (Kutch and Valero-Cuevas, 2012).

PCA is one of several methods to extract lower-dimensional representations of motor patterns (d'Avella et al., 2003; Ting and Macpherson, 2005; Clewley et al., 2008). Alternative techniques do not impose orthonormality constraints or over-estimate the real dimensionality of nonlinear underlying manifolds (Clewley et al., 2008). Similarly, Non-Negative Matrix Factorization (NMF) would not be subject to the flips in sign observed in Figure 5 (Tresch et al., 2006). We noted that for a given task intensity a muscle's activation across the sampled solutions can have different variance than the other muscles, and these variances change as task intensity increases (and the feasible activation space shrinks) (see the supplemental website for the task-variance figure). While PCA helps us uncover how these shapes change in this study, PCA can be leveraged to uncover different intramuscular relationships (e.g., analyzing the eigenvalue decomposition of the *correlation* matrix, as opposed to using PCA on the *covariance* matrix). Bootstrapping or data shuffling technique for sensitivity analysis are also applicable to dimensionality reduction techniques (Valero-Cuevas et al., 2016).

Feasibility Theory allows us to put dimensionality reduction in perspective. First, as a natural consequence of the definition of a task (i.e., the need to meet specific mechanical constraints). And second, as an approximation to the structure of the latent feasible activation space embedded in high-dimensions. While our results suggest caution when interpreting synergies obtained experimentally, we underscore that dimensionality reduction is, nevertheless, a useful approach to capture the general geometric properties of feasible activation spaces.

### Toward probabilistic neuromuscular control

Our results are particularly empowering for the emerging field of probabilistic neuromuscular control (Körding and Wolpert, 2004; Sanger, 2011; Kording, 2014). Suppose that the nervous system uses some form of probabilistic or Bayesian learning and control strategy. Such approach requires two enabling—and biologically plausible—elements: *trial-and-error iterative exploration* to build prior distributions, and *memory-based exploitation* of the probability density functions used to approximate the feasible activation spaces (Körding and Wolpert, 2004). The parallel coordinate plots and histograms in Figures 2, 7 provide, to our knowledge, the first complete (Smith, 1984; Lovász, 1999) characterization of such multi-dimensional conditional motor control spaces for a realistic tendon-driven system performing a well-defined task (i.e., activation of one muscle is contingent upon the activations of the other muscles). With a better understanding of the physical task, future studies into optimal motor control can leverage the feasible activation space to contextualize motor control policies, whether they are experimentally-observed or theoretically predicted (Berniker et al., 2013). As mentioned above, the muscle activation patterns that the nervous systems actually use will necessarily be a subset of these feasible activation spaces.

Feasibility Theory critically empowers the study of fundamental aspects of probabilistic control. For example, an organism can only execute so many trial-and-error iterations during learning, likely too few to completely and exhaustively sample the high-dimensional feasible space of interest. This makes it much more likely that, by virtue of being more easily found, an organism will find and preferentially exploit the strong modes (i.e., narrow and high peaks in Figures 3, 4, and 7) of the multi-dimensional probability density functions than any other region of feasible activation spaces. Thus, first, the maximal ranges of feasible activations described by the bounding box (Sohn et al., 2013; Valero-Cuevas et al., 2015b) may have little practical bearing on how those tasks are learned and executed. And second, those same strong modes would represent strong attractors to create and reinforce motor habits. Habitual control has been proposed based on experimental and empirical data as an alternative to a strict optimization approach to neuromuscular control (De Rugy et al., 2012; Fu et al., 2014). Our work now provides the computational means to link habitual to probabilistic control in isometric force production. This allows us to generate testable hypotheses of how these motor habits are defined by the structure of the feasible activation space, how easily they are learned by the organism, and how difficult or easy it is to break out of them (Raphael et al., 2010).

Motor function likely emerges from trial-and-error (Adolph et al., 2012) or imitation (Oztop et al., 2006; Cattaneo and Rizzolatti, 2009) to identify, remember and adopt easily-found, good enough solutions in the feasible activation space—independently of their cost. It is then possible to use some heuristic approach to improve performance to transition to less likely—but potentially “better” solutions as per some metric relevant to the individual—subregions of the solutions space. But this likely requires numerous iterations in practice, which explains why few are experts at a given motor task, or why rehabilitation is so difficult (Gladwell, 2008; Adolph et al., 2012; Lohse et al., 2014).

### Feasibility theory as a theory of motor control

Feasibility Theory goes beyond Bayesian control by underscoring how the *physics* of the body, and the properties of the task are the arbiter that guides the biological process of finding, exploring, inhabiting, and exploiting low-dimensional solution spaces embedded in high-dimensions. Feasibility Theory espouses heuristic local searches—driven by the memory of likelihoods of different individual solutions—to create what ultimately are useful, yet likely sub-optimal, motor habits. These processes hinge on trial-and-error, memory, pattern recognition, and reinforcement that come naturally to neural systems. Even though Feasibility Theory is presented in the context of neural control of the human hand, it applies to tendon-driven organisms in general.

Importantly, organisms perform strict optimization or synergy control at their peril. A feasible activation set is low-dimensional because it loses one dimension with each functional constraint that is being met (Valero-Cuevas et al., 1998; Inouye and Valero-Cuevas, 2016). Thus, moving along such low-dimensional spaces to find a new valid solution is equivalent to moving along a line (which has zero volume) in 3-dimensional space. Taking a step from any one valid point to another valid point on the feasible space runs the risk of “falling off” and failing at the task—a risk that is exponentially exacerbated in higher-dimensions. Thus, searching for improvements in the neighborhood of a known solution necessarily risk task failure and potential injury. These are all arguments in support of the evolutionary and developmentally useful strategy to use good-enough control based on habit or sensorimotor memory rather than optimization or synergy control (De Rugy et al., 2012; Fu and Santello, 2012).

This line of thinking has consequences to neurorehabilitation. Neurological conditions disrupt feasible activation spaces, be it by affecting anatomy of the limb, muscle strength, and independence with which muscles are controlled. Functional recovery following the disruption, if not destruction, of the landscape of valid muscle activation patterns, requires re-learning existent or building new probability density functions. Older adults suffering from reduced perceptuo-motor learning rates are presented an even more constrained feasibility space (Coats et al., 2014).

A probabilistic landscape for neuromuscular function begins to explain why neurorehabilitation in aging adults is so difficult (e.g., Lohse et al., 2014; Hardwick et al., 2016) and why motor learning in children takes thousands of repetitions (Adolph et al., 2012). But it empowers us to leverage knowledge of the families of feasible solutions to create new rehabilitation strategies and testable hypotheses around them.

## Data availability

The datasets generated and analyzed for this study can be found freely available (Git Repository Link), and at the supplemental website (Supplemental Site Link). We designed a web-based parallel coordinate visualization that lets users interactively limit muscles, select solutions, and calculate effects on the feasible activation space from each *post-hoc* constraint (Figure 4). Our companion site includes ample documentation, code implementation in Scala (with a comprehensive test suite), and all data visualization code in R, including an overhead view of Figure 7.

## Author contributions

BC: Study design, computational implementation, experimental analysis, and biological interpretation; MS and BG: Mathematical basis and composition of computational geometry techniques; FV-C: Study design, experimental analysis, theoretical and biological interpretation.

### Conflict of interest statement

The authors declare that the research was conducted in the absence of any commercial or financial relationships that could be construed as a potential conflict of interest.

## Footnotes

^1^Mechanical constraints is a formal way to call the physical demands, requirements, or characteristics of a given physical task.

^2^Neuromuscular control is variously referred to as, inter alia, neural, motor, sensorimotor control.
